# Hyperlipidemia and statins use for the risk of new-onset anxiety/depression in patients with head and neck cancer: A population-based study

**DOI:** 10.1371/journal.pone.0174574

**Published:** 2017-03-31

**Authors:** Chung-I Huang, Li-Ching Lin, Hung-Cheng Tien, Jenny Que, Wei Chen Ting, Po-Chun Chen, Hsin-Min Wu, Chung-Han Ho, Jhi-Joung Wang, Ren-Hong Wang, Ching-Chieh Yang

**Affiliations:** 1 Department of Radiation Oncology, E-Da Cancer Hospital, Kaohsiung, Taiwan; 2 Department of Radiation Oncology, Chi-Mei Medical Center, Tainan, Taiwan; 3 Department of Psychiatry, Pingtung Hospital, Ministry of Health and Welfare, Pingtung, Taiwan; 4 Department of Radiation Oncology, Pingtung Christian Hospital, Pingtung, Taiwan; 5 Department of Public Health, National Cheng Kung University Hospital, College of Medicine, National Cheng Kung University, Tainan, Taiwan; 6 Department of Medical Research, Chi-Mei Medical Center, Tainan, Taiwan; 7 Department of Pharmacy, Chia-Nan University of Pharmacy and Science, Tainan, Taiwan; 8 Department of Clinical Pathology, Chi-Mei Medical Center, Tainan, Taiwan; 9 Department of Medical Laboratory Science and Biotechnology, Chung Hwa University of Medical Technology, Tainan, Taiwan; 10 Institute of Biomedical Sciences, National Sun Yat-Sen University, Kaohsiung, Taiwan; 11 Department of Biotechnology, Chia-Nan University of Pharmacy and Science, Tainan, Taiwan; University of Cincinnati College of Medicine, UNITED STATES

## Abstract

**Objective:**

Anxiety/depression is common among patients with head and neck cancer (HNC), and can negatively affect treatment compliance and outcome. The aim of this study was to assess the association between hyperlipidemia and the risk of new-onset anxiety/depression after the diagnosis of HNC and the influence of administering statins.

**Methods:**

A matched longitudinal cohort study of 1632 subjects (408 HNC patients with preexisting hyperlipidemia and 1224 age- and sex-matched HNC patients without hyperlipidemia) was included and analyzed by using data from Taiwan’s National Health Insurance Research Database from January 1996 to December 2012. The incidence and hazard ratios (HRs) for the development of new-onset anxiety/depression were examined between the two groups. Cox proportional hazard regression was applied to estimate the relative risks of anxiety/depressive disorders adjusted for potential confounding factors. To estimate the risks of anxiety/depression in different sub-groups, a stratified analysis was also used.

**Results:**

HNC patients with preexisting hyperlipidemia had a higher risk for comorbidities such as hypertension, diabetes mellitus, and cardiovascular disease (*P* <0.001). The incidence rate of anxiety/depression in the HNC patients with preexisting hyperlipidemia was also significantly higher than that among patients without hyperlipidemia (10.78% vs 7.27%, respectively; *P* = 0.03). A Cox regression model revealed that preexisting hyperlipidemia was an independent risk factor for anxiety/depression (aHR, 1.96; 95% CI, 1.30–2.94). Statins use was protective against anxiety/depression among HNC patients with hyperlipidemia (aHR, 0.85; 95% CI, 0.46–1.57), especially for individuals older than 65 years and for females.

**Conclusions:**

Preexisting hyperlipidemia was associated with increased risk of new-onset anxiety/depression in the HNC patients. Statins use for HNC patients with hyperlipidemia could decrease the risk of anxiety/depression, especially for those older than 65 years and for female patients.

## Introduction

Head and neck cancer (HNC) is one of the most common malignancies across the globe. With its incidence rising, HNC has become the sixth most common type of cancer in Taiwan, and has been the fourth most common type of cancer among men since 2006 [1]. HNC patients are sometimes required to undergo distressing and disfiguring treatments, which often have a very high social and personal cost. As a result, HNC patients have the highest prevalence rates of psychological distress, such as depression or anxiety, of all cancer patients [2]. Too often, however, such emotional dysfunction is ignored and thus goes untreated [3, 4].

Although anxiety and depression are different disorders, they are both caused by a combination of multiple genetic and environmental factors [5]. Furthermore, patients often struggle with both disorders due to their overlapping symptoms and clinical presentation [6]. The effects of anxiety/depression can be especially severe for HNC patients, negatively affecting their quality of life and interfering with decision-making, treatment compliance, and outcome. These negative effects may also persist long after treatment ends [7]. Understanding the time course of psychological distress in HNC patients is important so that early intervention can take place.

Although causative factors associated with anxiety/depression have not been specifically analyzed for HNC patients, it has been hypothesized that they may be affected because of the multiple factors involved with the disease. Many of these patients have a history of substance abuse and also experience emotional distress due to the functional changes caused by treatment (impaired eating, speaking, taste, smell, and breathing) and the course of the illness itself [8]. A few recent studies have reported a correlation between hyperlipidemia and increased risk of anxiety [9] and depression [10]. Hyperlipidemia is a common symptom in the general population and is also strongly related to the risk of cardiovascular disease, diabetes mellitus, and hypertension, all risk factors for anxiety/depression [11, 12].

The 3-hydroxy-3-methylglutaryl-coenzyme A (HMG-CoA) reductase inhibitors (statins), a therapeutic class of drugs that reduce endogenous cholesterol levels, are used to manage and prevent coronary heart disease and stroke [13]. Several investigators have noted that statins users may have a lower risk of depression than nonusers [10]. However, it remains unclear how statins may benefit cancer patients, especially those with the characteristic HNC seen in Taiwan.

The primary aim of this study was to investigate the incidence of new-onset anxiety/depression in HNC patients with preexisting hyperlipidemia identified through Taiwan’s National Health Insurance Research Database (NHIRD). This study also allowed for a comparison of the risk of anxiety/depression between HNC patients with statins use and HNC patients who did not use statins. The results could provide an opportunity to demonstrate whether use of statins decreases the incidence of emotional dysfunction for HNC patients.

## Materials and methods

### NHIRD dataset and ethical considerations

This study used data from the NHIRD, which was released by the Taiwan National Health Research Institute (NHRI), and is available to all researchers in Taiwan. Taiwan initiated its National Health Insurance (NHI) program in March 1995. This system currently enrolls up to 99% of the Taiwanese population and contracts with 97% of all medical providers in Taiwan [14]. In this study, we used the Longitudinal Health Insurance Database 2000, which was released from the NHI organization, and included 1,000,000 randomly selected subjects, based on reimbursement data in 2000. The database contains comprehensive information on all insured individuals, including their diagnosis, age, gender, cancer type, comorbid diseases, socioeconomic status, any treatment given, medication use, and death. Information on tobacco use, dietary habits, and body mass index (BMI) were not included in this database. The database contained a registry of contracted medical facilities, a registry of board-certified physicians, and monthly medical insurance claims summaries for all inpatient claims. Disease diagnoses for all insured patients are classified according to the International Classification of Diseases, Ninth Revision, Clinical Modification (ICD-9-CM). This study was approved by the Institutional Review Board of Chi-Mei Medical Center, Tainan City, Taiwan. The usual review board requirements for written informed consent were waived because all personal identifying information was removed from the dataset prior to analysis.

### Population inclusion and exclusion criteria

Patients with diagnoses of HNC (ICD-9-CM: 140–149) made between 1996 and 2012 were identified from the NHIRD database. Among these patients, preexisting hyperlipidemia was defined as at least 3 outpatient visits within one year or 1 inpatient admission with ICD-9-CM coding 272.0, 272.1, 272.2, and 272.4 before the cancer was diagnosed. All healthcare services and patient information were followed for at least one year, until December 31, 2013, or until the patient’s death. New-onset anxiety/depression was based on the following ICD-9-CM coding: 295, 297, 300, 301, 296.2, 296.5, 311, 300.4, 296.82, 309.0, and 309.1. Exclusion criteria included: (1) hyperlipidemia noted after the diagnosis of HNC; (2) anxiety or depression detected before the diagnosis of HNC; or (3) age under 18 years. Other potential confounding factors, including different types of therapy (chemotherapy, radiotherapy, surgery), treatment with statins, comorbidities, hypertension (ICD-9-CM: 401–405), diabetes mellitus (ICD-9-CM: 250), and coronary artery disease (ICD-9-CM: 410–414), were also used for adjusting the effects of anxiety/depression among HNC patients. Subjects were matched according to age and gender, in a ratio of 1:3. A total of 1,632 subjects (408 HNC patients with preexisting hyperlipidemia and 1,224 age- and gender-matched HNC patients without hyperlipidemia) were analyzed in this study.

### Statistical analysis

To compare the differences between HNC patients with preexisting hyperlipidemia and those without, the Student's t-test was used for continuous variables and Pearson's Chi-square test or Fisher’s exact test was used for categorical variables. The Kaplan-Meier method was plotted to describe the probability of patients being free from anxiety/depressive disorders. The log-rank test was used to compare the risks between different groups. Cox proportional hazard regression was applied to estimate the relative risks of anxiety/depressive disorders, and was adjusted for potential confounding factors. The stratified analysis was also used to estimate the risk of anxiety/depression in different subgroups. All statistical analyses were performed with SAS 9.4 for Windows (SAS Institute, Inc, Cary, NC), and Kaplan-Meier curves were plotted from Stata software (Stata version 12; StataCorp LLC, College Station, TX). A *P*-value < 0.05 was considered significant.

## Results

**Fig 1:**
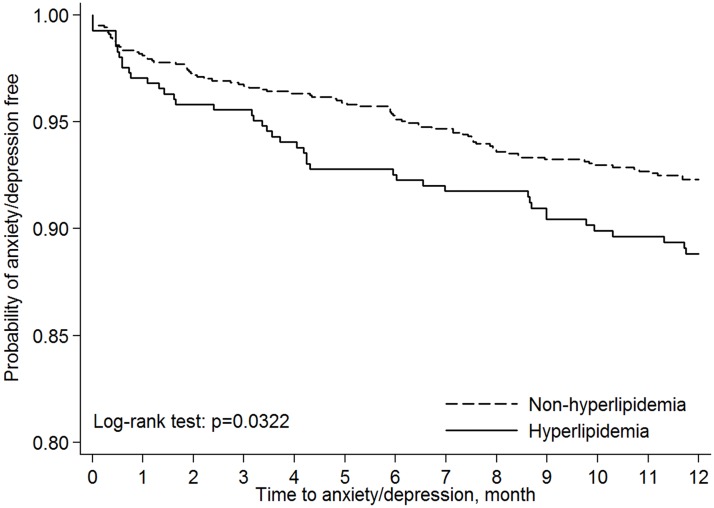
Kaplan-Meier probability for anxiety/depression-free status in head and neck cancer patients with and without hyperlipidemia.

A total of 1,632 patients were enrolled in this study. Table 1 shows the characteristics of HNC patients with and without hyperlipidemia. These two groups were matched by age and gender. There were significant differences in comorbidities between the patients with or without hyperlipidemia, but no significant differences were noted for cancer-related treatment. The incidence of anxiety/depression was 10.78% and 7.27% for the group with HNC with hyperlipidemia and the group without hyperlipidemia, respectively (*P* = 0.0282). The median time between diagnosis of HNC and new-onset anxiety/depression was similar between these two groups (3.5 and 4.31 months; *P* = 0.9809). The Kaplan-Meier plots (Fig 1) showed that HNC patients with hyperlipidemia had a significantly higher risk of anxiety/depression than did those without hyperlipidemia (log-rank test; *P* = 0.0322).

**Table 1:** Demographics and clinical characteristics of patients with head and neck cancer (HNC) with and without preexisting hyperlipidemia.

	HNC with Hyperlipidemia(N = 408)	HNC without Hyperlipidemia(N = 1224)	p-value^b^
Age (mean±SD)	58.04±11.38	58.05±11.38	0.9933
Age Group			
≦35	6(1.47)	18(1.47)	1.0000
36~50	90(22.06)	270(22.06)	
51~65	193(47.30)	579(47.30)	
>65	119(29.17)	357(29.17)	
Gender			
Male	356(87.25)	1068(87.25)	1.0000
Female	52(12.75)	156(12.75)	
Comorbidity			
HTN			
Yes	185(45.34)	158(12.91)	< .0001
No	223(54.66)	1066(87.09)	
DM			
Yes	165(40.44)	82(6.70)	< .0001
No	243(59.56)	1142(93.30)	
CAD			
Yes	58(14.22)	39(3.19)	< .0001
No	350(85.78)	1185(96.81)	
Treatment^a^			
Yes	296(72.55)	859(70.18)	0.3793
No	112(27.45)	365(29.82)	
Radiology therapy			
Yes	229(56.13)	641(52.37)	0.2075
No	179(43.87)	583(47.63)	
Chemical therapy			
Yes	180(44.12)	527(43.06)	0.7294
No	228(55.88)	697(56.94)	
Surgery therapy			
Yes	125(30.64)	392(32.03)	0.6234
No	283(69.36)	832(67.97)	
Outcome			
Anxiety/depression			
Yes	44(10.78)	89(7.27)	0.0282
No	364(98.22)	1135(92.73)	
Time to anxiety/ depression (month), median(IQR)	3.50(0.74–7.80)	4.31(0.99–7.43)	0.9809

^a.^ Treatment: Radiology therapy, Chemical therapy, Surgery therapy

^b.^ p-value is from the Chi-squared test or Fisher’s exact test for categorical variables

After adjusting for age, gender, comorbidities, and cancer-related treatments, the incidence of anxiety/depression remained higher among those with than among those without preexisting hyperlipidemia (aHR = 1.96; 95% CI, 1.30–2.94; Table 2). In addition, patients who had received cancer treatment, including surgery, chemotherapy, and radiotherapy, for HNC had a lower risk for anxiety/depression than did patients who did not receive treatment (aHR = 0.60; 95% CI, 0.42–0.86).

**Table 2:** Cox proportional hazard regressions of patients with head and neck cancer (HNC) with and without hyperlipidemia.

Variable	Crude HR (95% CI)	p-value	Adjusted HR (95% CI)	p-value
**Hyperlipidemia**				
No Hyperlipidemia	Ref		Ref	
Hyperlipidemia	1.48(1.03–2.12)	0.0333	1.96(1.30–2.94)	0.0012
**Age Group**				
≦35	Ref		Ref	
36~50	0.59(0.21–1.67)	0.3234	0.68(0.24–1.92)	0.4657
51~65	0.47(0.17–1.29)	0.1409	0.57(0.21–1.58)	0.2814
>65	0.45(0.16–1.27)	0.1307	0.55(0.19–1.57)	0.2659
**Gender**				
Male	Ref		Ref	
Female	1.02(0.61–1.70)	0.9333	0.95(0.57–1.60)	0.8581
**Comorbidity**				
HTN	0.75(0.48–1.19)	0.2234	0.70(0.42–1.17)	0.1751
DM	0.77(0.46–1.30)	0.3306	0.64(0.36–1.14)	0.1319
CAD	0.76(0.33–1.71)	0.5033	0.79(0.33–1.87)	0.5944
**Treatment**^a^				
No	Ref		Ref	
Yes	0.63(0.45–0.90)	0.0105	0.60(0.42–0.86)	0.0049

^a.^ Treatment: Radiology therapy, Chemical therapy, Surgery therapy

**Fig 2:**
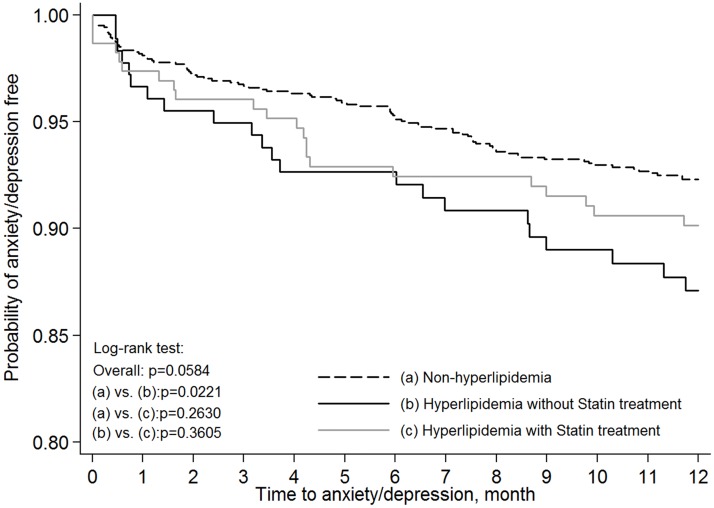
Kaplan-Meier probability for anxiety/depression-free status in head and neck cancer patients without preexisting hyperlipidemia and with hyperlipidemia treated or not treated with statins.

We further analyzed the impact of using statins on the risk of anxiety/depression in HNC patients with preexisting hyperlipidemia. Table 3 shows the distribution of patients who used statins and those who did not. These two groups were significantly different in age, comorbidities, and treatment. Fig 2 demonstrates the Kaplan-Meier plots of anxiety/depression free probability for the HNC patients without hyperlipidemia and for patients with preexisting hyperlipidemia treated with or without statins. A comparison of those groups showed that the patients with hyperlipidemia without statins treatment had significantly higher risk of anxiety/depression compared to patients without hyperlipidemia (*P* = 0.0221). On the other hand, those patients with hyperlipidemia treated with statins did not have significantly higher risk of anxiety/depression compared to those without hyperlipidemia (*P* = 0.2630). However, there was no significant difference when directly comparing the hyperlipidemia patients treated with statins to those not treated with statins (*P* = 0.3605). This might explain why the overall analysis of the HNC patients with preexisting hyperlipidemia treated with statins only showed a trend toward a protective effect against anxiety/depression compared to the hyperlipidemia patients who did not use statins (*P* = 0.0584). After stratification with differing variables, such as age, gender, comorbidities, and treatment, we observed that statins use played a protective role against anxiety/depression (aHR = 0.85; 95% CI, 0.46–1.57; Table 4). Notably, age and gender also influenced the results. For female patients or patients older than 65 years with preexisting hyperlipidemia, there was a protective effect with statins use (aHR = 4.65; 95%CI, 1.33–16.27 for females; aHR = 3.7; 95% CI, 1.49–9.21 for patients older than 65 years) compared with those without statins use (aHR = 5.34, 95% CI, 1.56–18.34 for females; aHR = 4.15, 95%CI, 1.66–10.40 for patients older than 65 years).

**Table 3:** The distribution of patients with head and neck cancer (HNC) with hyperlipidemia between statin treatment or not.

	HNC with Hyperlipidemia with statin treatment (N = 228)	HNC with Hyperlipidemia without statin treatment(N = 180)	p-value^b^
Age Group			
≦35	1(0.44)	5(2.78)	0.0165
36~50	41(17.98)	49(27.22)	
51~65	119(52.19)	74(41.11)	
>65	67(29.39)	52(28.89)	
Gender			
Male	197(86.40)	159(88.33)	0.6543
Female	31(13.60)	21(11.67)	
Comorbidity			
HTN			
Yes	125(54.82)	60(33.33)	< .0001
No	103(45.18)	120(66.67)	
DM			
Yes	103(45.18)	62(34.44)	0.0329
No	125(54.82)	118(65.56)	
CAD			
Yes	40(17.54)	18(10.00)	0.0326
No	188(82.46)	162(90.00)	
Treatment			
Yes	155(67.98)	141(78.33)	0.0252
No	73(32.02)	39(21.67)	
Radiology therapy			
Yes	121(53.07)	108(60.00)	0.1915
No	107(46.93)	72940.00)	
Chemical therapy			
Yes	85(37.28)	95(52.78)	0.0019
No	143(62.72)	85(47.22)	
Surgery therapy			
Yes	68(29.82)	57(31.67)	0.7458
No	160(70.18)	123(68.33)	
Outcome			
Anxiety/depression			
Yes	22(9.65)	22(12.22)	0.4252
No	206(90.35)	158(87.78)	

^b.^ *p*-value is from the Chi-squared test or Fisher’s exact test for categorical variables

**Table 4:** Stratified analysis for hazard ratio in different variables among patients with head and neck cancer (HNC) without preexisting hyperlipidemia and with hyperlipidemia treated or not treated with Statins.

Variable	Non-Hyperlipidemia	HNC with Hyperlipidemia without statin treatment	HNC with Hyperlipidemia with statin treatment
**Overall**			
All study subjects	Ref	2.12(1.30–3.47)*	1.79(1.06–3.02)
Case only		Ref	0.85(0.46–1.57)
**Age** ≦35			
All study subjects	Ref	1.14(0.11–11.58)	—
Case only		Ref	—
**Age**: 36~50			
All study subjects	Ref	1.20(0.41–3.52)	2.23(0.85–5.83)
Case only		Ref	1.86(0.50–6.96)
**Age**: 51~65			
All study subjects	Ref	2.16(1.04–4.49)*	1.04(0.44–2.47)
Case only		Ref	0.50(0.19–1.35)
**Age** >65			
All study subjects	Ref	4.15(1.66–10.40)*	3.70(1.49–9.21)*
Case only		Ref	0.88(0.32–2.44)
**Males**			
All study subjects	Ref	1.79(1.04–3.08)*	1.44(0.80–2.58)
Case only		Ref	0.76(0.38–1.53)
**Females**			
All study subjects	Ref	5.34(1.56–18.34)*	4.65(1.33–16.27)*
Case only		Ref	2.96(0.35–24.74)
**Comorbidity: HTN**			
All study subjects	Ref	0.93(0.28–3.11)	0.85(0.31–2.31)
Case only		Ref	0.95(0.28–3.20)
**Comorbidity: DM**			
All study subjects	Ref	0.77(0.21–2.88)	0.52(0.16–1.68)
Case only		Ref	0.68(0.19–2.51)
**Comorbidity: CAD**			
All study subjects	Ref	1.24(0.07–23.53)	2.00(0.20–19.99)
Case only		Ref	1.93(0.20–18.56)
**Treatment**^a^			
All study subjects	Ref	1.97(1.08–3.58)*	1.44(0.71–2.93)
Case only		Ref	0.67(0.30–1.51)

*p-value<0.05

^a.^ Treatment including Radiology therapy, Chemical therapy, Surgery therapy

## Discussion

The results of this study demonstrated that the risk of new onset of anxiety/depression for patients with HNC was independently influenced by preexisting hyperlipidemia. There was a 1.96-fold increased risk of anxiety/depression in HNC patients with hyperlipidemia compared to those without hyperlipidemia. Statins use could decrease the risk of anxiety/depression for the HNC patients with preexisting hyperlipidemia, particularly among older adults (>65 years) and among women. This information will hopefully serve as a foundation for future studies to improve emotional dysfunction in HNC patients.

One major strength of this study was the use of the nationwide population-based NHIRD of Taiwan, which is a record of actual medical practice, including comprehensive information about clinical care. Moreover, the NHIRD records contained complete follow-up information, including all disease events, for the entire study population. These records show all healthcare benefits with a moderate cost-sharing, and also reveal regular monitoring of diagnostic accuracy and treatment. As we know, randomized controlled trials or meta-analysis review studies are the gold standard in clinical research and also provide the strongest evidence for resolving clinical problems [15]. Population-based observation studies with large case numbers are also useful. First, such studies can delineate what has been achieved in the real clinical world [16, 17]. Second, they enable researchers to explore an association of rare events. And, finally, they make it possible to approach issues that are difficult or not feasible for investigation with randomized controlled trials within a limited period [16].

For cancer patients, anxiety and depression frequently arrive together and should be taken seriously. Although anxiety and depression have been widespread among cancer patients (a prevalence of 76% in one report), they are usually ignored and untreated [3]. Furthermore, anxiety and depression are also associated with poor prognosis [18]. While caring for cancer patients, we must pay attention to early detection of and intervention for psychological distress. For HNC patients, who may have problems stemming from the withdrawal syndrome, physical disfigurement, side effects from treatment, or disease progression and challenges with social contacts, the prevalence of depressive symptoms has always been reported among the highest rates within the entire cancer population [2, 19].

The results of some studies have shown that the prevalence rates of depressive symptoms are affected by factors such as age, sex, comorbidities, urban life, socioeconomic status, and type of cancer [20]. Few studies have examined the relationship between hyperlipidemia and depressive or anxiety disorders [21, 22]. Dutch researchers compared levels of serum total, low-density lipid, and high-density lipid cholesterol and triglycerides among 761 major depressive disorder (MDD) patients, 1071 remitted MDD subjects, and 629 controls. The results showed lower HDL cholesterol (*P* = 0.007) and triglyceride levels (*P* = 0.001) in the MDD group compared with the remitted MDD group and controls [23]. A population-based study in Taiwan also revealed similar results. Chuang et al. analyzed 134,260 subjects from the NHI Database in Taiwan [10]. Among those subjects, 26,825 patients with newly diagnosed hyperlipidemia had an increased incidence of depression (HR, 1.64; 95% CI, 1.55–1.74) compared to those without hyperlipidemia. This observation was similar to our findings: in our study, patients with preexisting hyperlipidemia had a significantly higher risk of new-onset anxiety/depression (10.78% vs 7.27%; *P* = 0.03).

Considering the association between hyperlipidemia and psychological distress, there should be certain pathways involving this phenomenon. Hyperlipidemia is believed to be associated with elevated levels of systemic inflammation [24]. Systemic inflammation and inflammatory mediators precipitate development of atherosclerosis and other vascular changes, and ischemic lesions that might correlate to depressive symptoms [25]. Martinac et al. hypothesized that metabolic syndrome and depressive disorder were connected through hyperactivity of the hypothalamic-pituitary-adrenal (HPA) axis and changes in the immune system [26]. Future studies are expected to explore possible mechanisms involved with the occurrence of depressive syndromes, which are still not well understood.

Meanwhile, statins are commonly prescribed to treat hyperlipidemia. These agents have proved to be effective for reducing plasma lipid levels and for preventing cardiovascular disease [27]. When Can et al. examined the effect of simvastatin in rats, they found that a high-fat diet consumed through the prenatal and postnatal periods increased serum triglyceride levels, enhanced anxiety and depression, and reduced cognitive performance [28]. However, the negative effects of the high-fat diet were reversed after simvastatin treatment. This anxiolytic or antidepressant-like effect of statins might be attributed to modulation of endothelial function and reduced inflammatory processes [27]. In addition, the statins may counteract the negative effects of hyperlipidemia as a consequence of lowering serum lipid levels. Another factor is that not only could lipophilic statins (e.g., atorvastatin, lovastatin, fluvastatin, pitavastatin, simvastatin) cross the brain-blood barrier, but hydrophilic statins could also enter the neuroparenchyma. This might be a possible mechanism for statins to affect cognitive function, neurodegenerative disease, and various neurological disorders, such as stroke, epilepsy, depression, and central nervous system cancers [29]. A few studies have reported negative or inconclusive effects of statins on mood [30, 31]. One researcher even reported that lower serum cholesterol levels are associated with increased mortality from suicide [32]. But, in a recent meta-analysis study, O'Neil et al. reviewed 7 randomized controlled trials involving 2,105 participants (1,133 patients treated with simvastatin, atorvastatin, fluvastatin, lovastatin, mevastatin, pitavastatin, pravastatin, rosuvastatin, or cerivastatin, and 972 subjects in a placebo group) [33]. Patients treated with statins had significantly improved mood scores (standardized mean difference -0.43; 95% CI, –0.61 to -0.24). The authors concluded that the results support mood-related benefits from statins use.

In brief, the results of these studies support the premise that statins could lower the incidence of new-onset anxiety and depression in patients with hyperlipidemia. However, in our analysis, there was a trend of statistical significance for the statins against anxiety/depression for HNC patients who already had hyperlipidemia. In stratified analysis, statins use could be protective against anxiety/depression for female patients or patients older than 65 years with preexisting hyperlipidemia (aHR = 3.7; 95% CI, 1.49–9.21; aHR = 4.65; 95% CI, 1.33–16.27).

It is worth mentioning that in our study nearly 30% of patients (27.45% and 29.82%) did not receive any treatment for their HNC. That percentage seems remarkably high, and there are some possible reasons for this. The first explanation for the high non-treatment rate might be due to a higher mortality in this study population. If mortality was defined as in-hospital deaths, the mortality rate may be lower. In our study, the one-year in-hospital mortality for non-treated patients was 8 among HNC patients with hyperlipidemia (7.14%, time to death: 4.03±4.72) and 38 in HNC patients without hyperlipidemia (10.41%, time to death: 3.59±3.70). Another consideration is that some patients with advanced-stage cancer delay seeking proper in-hospital medical treatment and seek alternative therapy after the diagnosis or in some cases receive only palliative care.

### Study limitations

To our knowledge, this is the first study to focus on the influence of hyperlipidemia and statins use in the onset of anxiety/depression among HNC patients. However, this population-based study did have some limitations. First, we could not directly check for statins use in these HNC patients. We presumed that all medications were actually taken as prescribed. Overestimation of the actual ingested dosage may occur due to some degree of noncompliance. Furthermore, in this study, the dosage, total treatment length, and comparison of the effects of different kinds of statins use were not available. These factors should be investigated in our future research. Another limitation was that hyperlipidemia was defined as patients coded with the ICD-9-CM. The personal habits, dietary habits, BMI, and lipid profiles, which may influence the development of anxiety/depression disorder, are not provided in the NHIRD. Thus, these potential contributors to our results may be undetected. Another factor is that treatment for cancer is a long-term process. And the onset of anxiety/depression in these cancer patients might also be much more affected by the status of cancer, e.g., the stage of the disease, tumor response to the treatment, and recurrence or metastases. Regretfully the NHIRD did not code the cancer status. A degree of early-onset anxiety and depression might have occurred shortly after diagnosis and before any treatment was given. Hence, even though our data showed that patients receiving treatment had a lower risk of anxiety/depression, some bias could have occurred. Finally, the patients in this study group consisted of up to 99% Taiwanese residents, who are mostly Asian. Racial variations are known to affect lipid profiles. Because of the lack of patient data for Caucasians and other ethnicities, our results should be further validated in patients in Western countries.

## Conclusions

Preexisting hyperlipidemia was associated with increased risk of new-onset anxiety/depression in HNC patients. Statins use for HNC patients with preexisting hyperlipidemia could decrease the risk of anxiety/depression, especially for patients older than 65 years and for female patients. Future clinical studies or randomized controlled trials might be required to confirm the benefits of statins use, which can improve the incidence of emotional dysfunction in HNC patients.

## Abbreviations

BMI: body mass index
CI: confidence interval
HDL: high-density lipoprotein
HNC: head and neck cancer
HR: hazard ratio
ICD-9CM: International Classification of Diseases, Ninth Revision, Clinical Modification
LDL: low-density lipoprotein
MDD: major depressive disorder
NHIRD: National Health Insurance Research Database
